# Reports of COVID-19 Vaccine Adverse Events in Predominantly Republican vs Democratic States

**DOI:** 10.1001/jamanetworkopen.2024.4177

**Published:** 2024-03-29

**Authors:** David A. Asch, Chongliang Luo, Yong Chen

**Affiliations:** 1 Division of General Internal Medicine, University of Pennsylvania, Philadelphia; 2 Leonard Davis Institute of Health Economics, University of Pennsylvania, Philadelphia; 3 Division of Public Health Sciences, Washington University School of Medicine in St Louis, St Louis, Missouri; 4 Center for Health AI, University of Pennsylvania, Philadelphia; 5 Department of Biostatistics, Epidemiology, and Informatics, University of Pennsylvania, Philadelphia

## Abstract

**Importance:**

Antivaccine sentiment is increasingly associated with conservative political positions. Republican-inclined states exhibit lower COVID-19 vaccination rates, but the association between political inclination and reported vaccine adverse events (AEs) is unexplored.

**Objective:**

To assess whether there is an association between state political inclination and the reporting rates of COVID-19 vaccine AEs.

**Design, Setting, and Participants:**

This cross-sectional study used the AE reports after COVID-19 vaccination from the Vaccine Adverse Event Reporting System (VAERS) database from 2020 to 2022, with reports after influenza vaccines from 2019 to 2022 used as a reference. These reports were examined against state-level percentage of Republican votes in the 2020 US presidential election.

**Exposure:**

State-level percentage of Republican votes in the 2020 US presidential election.

**Main Outcomes and Measures:**

Rates of any AE among COVID-19 vaccine recipients, rates of any severe AE among vaccine recipients, and the proportion of AEs reported as severe.

**Results:**

A total of 620 456 AE reports (mean [SD] age of vaccine recipients, 51.8 [17.6] years; 435 797 reports from women [70.2%]; a vaccine recipient could potentially file more than 1 report, so reports are not necessarily from unique individuals) for COVID-19 vaccination were identified from the VAERS database. Significant associations between state political inclination and state AE reporting were observed for all 3 outcomes: a 10% increase in Republican voting was associated with increased odds of AE reports (odds ratio [OR], 1.05; 95% CI, 1.05-1.05; *P* < .001), severe AE reports (OR, 1.25; 95% CI, 1.24-1.26; *P* < .001), and the proportion of AEs reported as severe (OR, 1.21; 95% CI, 1.20-1.22; *P* < .001). These associations were seen across all age strata in stratified analyses and were more pronounced among older subpopulations.

**Conclusions and Relevance:**

This cross-sectional study found that the more states were inclined to vote Republican, the more likely their vaccine recipients or their clinicians reported COVID-19 vaccine AEs. These results suggest that either the perception of vaccine AEs or the motivation to report them was associated with political inclination.

## Key Points

**Question:**

Is state political inclination associated with COVID-19 vaccine adverse event (AE) reporting?

**Findings:**

This cross-sectional study of 620 456 AE reports found that a 10% increase in state Republican voting was associated with a 5% increase in the odds that a COVID-19 vaccine AE would be reported, a 25% increase in the odds that a severe AE would be reported, and a 21% increase in the odds that any reported AE would be severe.

**Meaning:**

The findings suggest that the more states are inclined to vote Republican, the more likely their vaccine recipients or their clinicians are to report COVID-19 vaccine AEs.

## Teaser

This cross-sectional study assesses whether there is an association between state political inclination and the reporting rates of COVID-19 vaccine adverse effects.

## Introduction

COVID-19 mortality has been higher in US jurisdictions that are more conservative in their party registration,^1^ voting history,^2^ or representation.^3^ These differences are likely explained, in part, by differences in vaccination rates. Counties voting for former President Donald Trump in the 2020 presidential election had substantially lower COVID-19 vaccination rates than counties voting for President Joe Biden.^4,5^

Although antivaccine sentiment may originate from libertarian inclinations, the language communicating and reinforcing this sentiment typically invokes scientific evidence, reflecting lower confidence in vaccine effectiveness or greater concern about adverse events (AEs). To quantify the latter, we performed an ecological cross-sectional study of the association of state-level vaccine AE reporting for COVID-19 vaccines with state-level voting in the 2020 presidential election. We used the Vaccine Adverse Event Reporting System (VAERS), a surveillance system established in 1990 by the US Food and Drug Administration and the Centers for Disease Control and Prevention.^6^ Patients, clinicians, or manufacturers can report to the VAERS any symptoms plausibly associated with vaccination. Although the reporting format is standardized, reporting itself is voluntary, creating large risks of reporting bias.^7^

However, what is a weakness in the VAERS in systematically capturing AEs is a strength in quantifying the perception of AEs and the motivation to report them. Without a plausible reason to believe that vaccine recipients and their clinicians in Republican-inclined states will objectively encounter different rates of vaccine AEs than those in Democrat-inclined states, or have different abilities to report them, differences in reporting of those AEs will reflect the product of how those AEs are perceived and the inclination to report them, either by the vaccine recipients or their clinicians.

## Methods

This cross-sectional study examined the association between each state’s percentage of votes for the 2020 Republican presidential candidate with state-level reporting of COVID-19 vaccine AEs. We used the 2019 to 2022 (2020-2022 for COVID-19 and 2019-2022 for influenza vaccines) VAERS AE reports for adults in all 50 states and the District of Columbia. Adverse events were reported to the VAERS as severe if they threatened or caused death or led to emergency visits, hospitalizations, or disability.^8,9^ We included reports from men and women aged 18 years or older and excluded reports with no AE symptoms (often reports of product storage errors), reports when multiple vaccines were administered at the same time, or reports outside the 50 US states and District of Columbia. State population and election data sources and accession dates are listed and summarized in eTable 1 and eAppendix 1 in Supplement 1. The study was deemed exempt by the institutional review board of the University of Pennsylvania because the data were publicly available and not personally identifiable. This study followed the Strengthening the Reporting of Observational Studies in Epidemiology (STROBE) reporting guideline.

**Figure 1.**
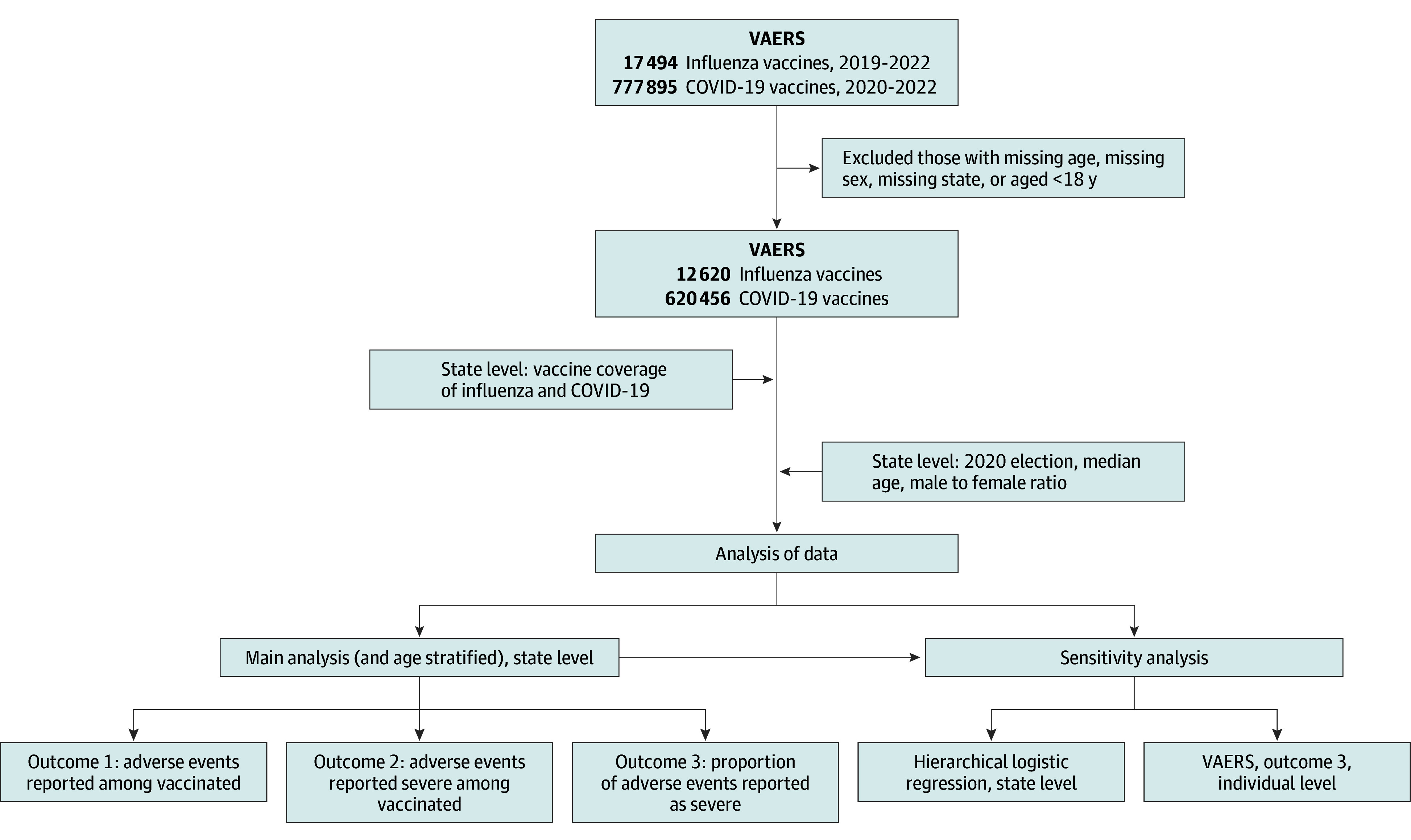
Flowchart of the Data Processing and Analysis Procedure VAERS indicates Vaccine Adverse Event Reporting System.

We separately examined 3 different outcomes: (1) rates of any AE among vaccine recipients, (2) rates of any severe AE among vaccine recipients, and (3) the proportion of AEs reported as severe. To account for baseline variation in VAERS reporting behavior across states, we also used each state’s AE reporting rate for the influenza vaccine (Figure 1).

For each of the 3 reporting rates, we developed a logistic regression model of counts of AE reports on the percentage of 2020 Republican votes. We included adjustments for the male to female ratio and median age and included 2019-2022 reporting of influenza vaccine AEs to adjust for state-level heterogeneity in VAERS reporting unconnected to COVID-19 vaccination. In sensitivity analyses, we (1) stratified regressions by age groups or excluded the District of Columbia; (2) used hierarchical logistic regression with state-specific random effects as another approach to account for heterogeneity in the baseline VAERS reporting rate; (3) performed an individual-level analysis for the proportion of AEs reported as severe within VAERS reports, adjusting for individual age, sex, and history of medication or allergy; and (4) relaxed assumptions of linearity by using locally estimated scatterplot smoothing.^10^ All *P* values were from 2-sided tests and results were deemed statistically significant at *P* < .05. Model specifications are detailed in eAppendix 2 in Supplement 1, and the processed data and analysis code in R software are available online.^11^

## Results

**Figure 2.**
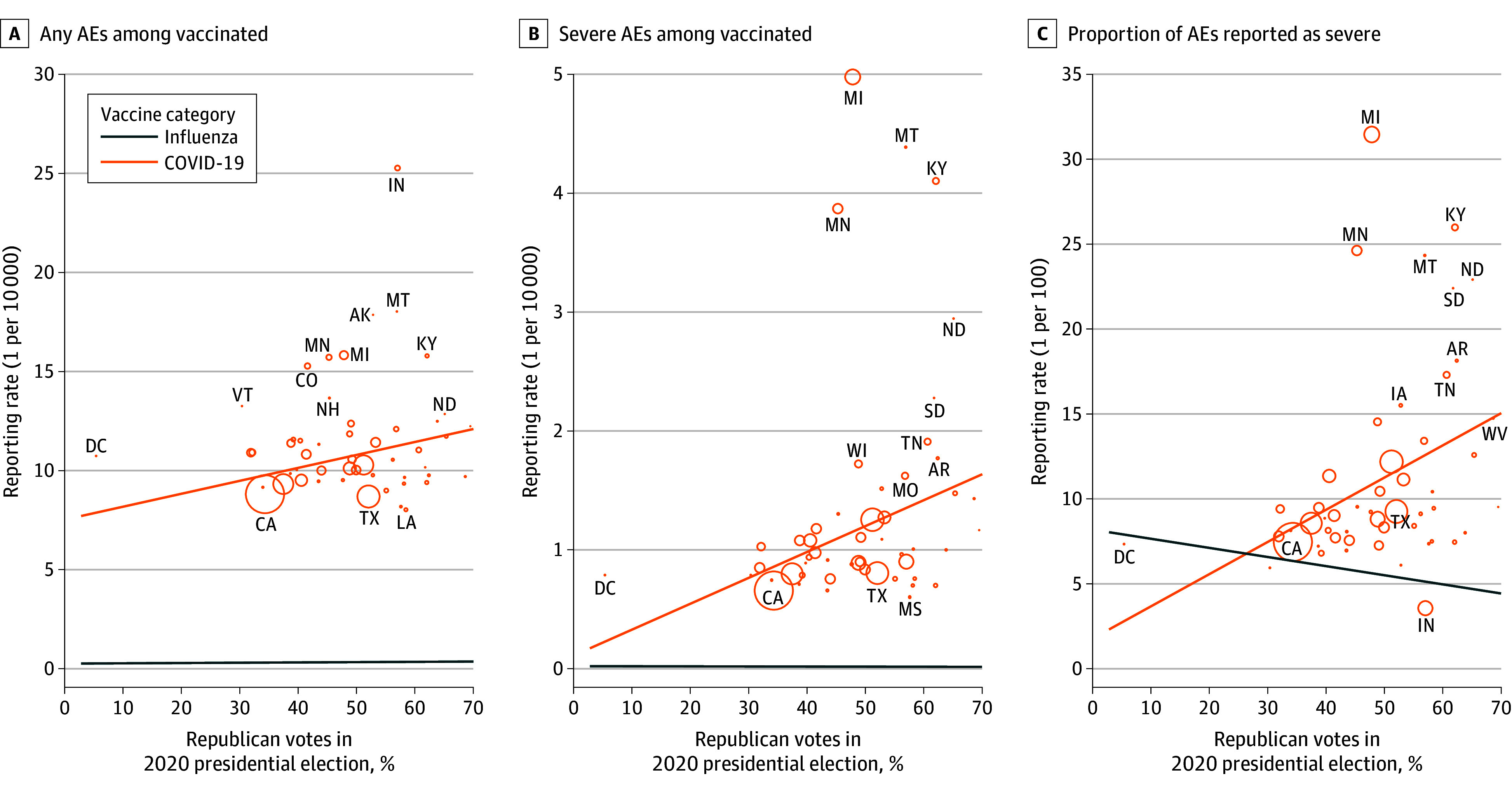
Adjusted Association Between State-Level Vaccine Adverse Event (AE) Reporting Rates and Political Inclination The 3 panels show state-level vaccine AE reporting for COVID-19 vaccines and influenza vaccines for (A) any AEs, (B) severe AEs, and (C) the proportion of AEs reported as severe. All associations with COVID-19 vaccines were positive and statistically significant; slopes for the orange lines are 6.5 per 1 000 000 vaccinated and 1% Republican (*P* = .001; *R*^2^ = 0.11), 2.2 per 1 000 000 vaccinated and 1% Republican (*P* < .001; *R*^2^ = 0.09), and 1.9 per 1000 reported and 1% Republican (*P* < .001; *R*^2^ = 0.07), respectively. The associations with influenza vaccines were represented by the slopes of the gray lines: 0.1 per 1 000 000 vaccinated and 1% Republican (*P* = .07; *R*^2^ = 0.23), −0.01 per 1 000 000 vaccinated and 1% Republican (*P* = .06; *R*^2^ = 0.08), and −0.54 per 1000 reported and 1% Republican (*P* = .004; *R*^2^ = 0.07), respectively. Circle sizes are proportional to the number of Vaccine Adverse Event Reporting System reports in the state. The circles for influenza vaccines are omitted for visual clarity.

**Figure 3.**
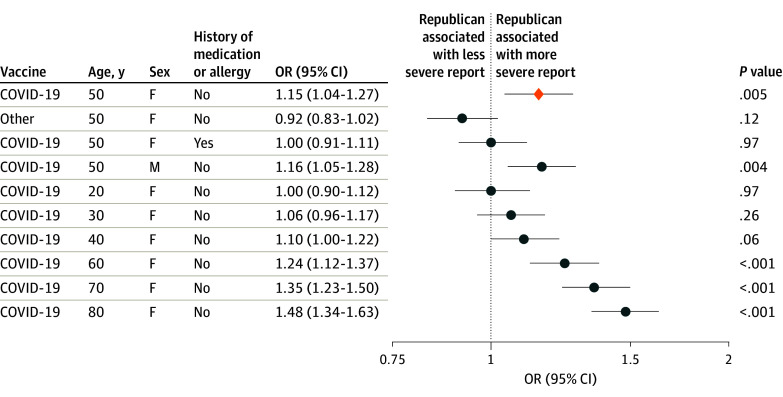
Second Sensitivity Analysis for the Association of Political Inclination With Severe Vaccine Adverse Event Reports Individual-level analysis of 620 456 reports associated with COVID-19 vaccines and 62 581 reports associated with any other vaccines within the Vaccine Adverse Event Reporting System. Each row represents a subgroup of recipients (eg, the first row is the subpopulation of women aged 50 years, without a history of medication or allergy). The first row shows that the association of political inclination is significant (OR, 1.15; 95% CI, 1.04-1.27; *P* = .005) for the baseline group (ie, COVID-19 vaccine, 50 years of age, and female with no history of medication or allergy). The other rows show that at baseline, a change in other factors (ie, potential association modifiers) may change the association of political inclination. For example, individuals with other vaccines or history of medication or allergy do not show a significant association with political inclination, male repcipients of the COVID-19 vaccine show a similar association with political inclination as female recipients, and older recipients of the COVID-19 vaccine show a stronger association with political inclination. “Other” indicates vaccines other than the COVID-19 vaccine.

We observed 620 456 AE reports (435 797 from women [70.2%]; mean [SD] age, 51.8 [17.7] years) associated with COVID-19 vaccination (Table 1). A vaccine recipient could potentially file more than 1 report, so reports are not necessarily from unique individuals. A 10% increase in state-level Republican voting was associated with increased odds of AE reports (odds ratio [OR], 1.05; 95% CI, 1.05-1.05; *P* < .001), severe AE reports (OR, 1.25; 95% CI, 1.24-1.26; *P* < .001), and the proportion of AEs reported as severe (OR, 1.21; 95% CI, 1.20-1.22; *P* < .001) (Table 2). These positive associations between political inclination and reports of COVID-19 vaccination AEs are shown in Figure 2 against no associations between political inclination and reports of influenza AEs. These findings were seen across all age strata in stratified analyses and in analyses excluding the District of Columbia (Table 2) and were sustained in sensitivity analyses (eTable 2 and the eFigure in Supplement 1; Figure 3).

**Table 1.** Distribution of Political Views (State Level), Age, Sex, and History of Medication or Allergy by Report Severity in VAERS Database

Factor	Nonsevere reports (n = 551 937)	Severe reports (n = 68 519)	Total reports (N = 620 456)	*P* value^a^
**COVID-19 vaccines (2020-2022)**				
Votes for Republican candidate, mean (SD), %	46.3 (9.2)	47.8 (8.8)	46.4 (9.2)	<.001
Candidate elected, No. (%)				
Democrat	339 297 (61.5)	41 673 (60.8)	380 970 (61.4)	<.001
Republican	212 640 (38.5)	26 846 (39.2)	239 486 (38.6)	
Age, mean (SD), y	50.6 (17.2)	61.9 (18.4)	51.8 (17.6)	<.001
Sex, No. (%)				
Female	399 172 (72.3)	36 625 (53.5)	435 797 (70.2)	<.001
Male	152 765 (27.7)	31 894 (46.5)	184 659 (29.8)	
**Influenza vaccines (2019-2022)**				
No.	11 911	709	12 620	
Votes for Republican candidate, mean (SD), %	46.9 (9.1)	46.1 (9.2)	46.9 (9.1)	.02
Candidate elected, No. (%)				
Democrat	7070 (59.4)	438 (61.8)	7508 (59.5)	.22
Republican	4841 (40.6)	271 (38.2)	5112 (40.5)	
Age, mean (SD), y	55.6 (18.1)	57.9 (18.3)	55.7 (18.1)	.001
Sex, No. (%)				
Female	8999 (75.6)	469 (66.1)	9468 (75.0)	<.001
Male	2912 (24.4)	240 (33.9)	3152 (25.0)	

Abbreviation: VAERS, Vaccine Adverse Event Reporting System.

^a^ Calculated by 2-sample *t* tests (for votes for Republican candidate and age) or χ^2^ tests (for candidate elected and sex).

**Table 2.** State-Level Association Analyses Between Political Inclination and COVID-19 Vaccine Adverse Event Reporting Rate

Age strata and outcome^a^	Odds ratio (95% CI)^b^	*P* value
Overall (aged ≥18 y)		
Adverse events among vaccinated individuals	1.05 (1.05-1.05)	<.001
Severe adverse events among vaccinated individuals	1.25 (1.24-1.26)	<.001
Proportion of severe adverse events	1.21 (1.20-1.22)	<.001
Aged 18-49 y		
Adverse events among vaccinated individuals	1.03 (1.03-1.04)	<.001
Severe adverse events among vaccinated individuals	1.10 (1.08-1.11)	<.001
Proportion of severe adverse events	1.02 (1.00-1.04)	.04
Aged 50-64 y		
Adverse events among vaccinated individuals	1.07 (1.06-1.07)	<.001
Severe adverse events among vaccinated individuals	1.19 (1.17-1.21)	<.001
Proportion of severe adverse events	1.11 (1.09-1.13)	<.001
Aged ≥65 y		
Adverse events among vaccinated individuals	1.05 (1.05-1.06)	<.001
Severe adverse events among vaccinated individuals	1.35 (1.33-1.36)	<.001
Proportion of severe adverse events	1.34 (1.33-1.36)	<.001
Overall (aged ≥18 y), excluding the District of Columbia		
Adverse events among vaccinated individuals	1.06 (1.05-1.06)	<.001
Severe adverse events among vaccinated individuals	1.26 (1.25-1.27)	<.001
Proportion of severe adverse events	1.22 (1.21-1.23)	<.001

^a^ The regression is fitted using overall data and age-stratified data and excluding the District of Columbia. State median age was not adjusted in age-stratified analyses.

^b^ Estimated from the state-level logistic regression model adjusting for state influenza vaccines reporting rates, male to female ratios, and median age.

## Discussion

This study found that the more states were inclined to vote Republican in the 2020 US presidential election, the more likely their vaccine recipients or clinicians were to report COVID-19 vaccine AEs. This association between political inclination and vaccine AE reporting was not seen for the influenza vaccine. The results are consistent with a relative overreporting of vaccine AEs among Republicans or a relative underreporting among Democrats.

### Limitations and Strengths

This study is limited by its ecological design.^12^ Both vaccine reporting and political voting occur at the level of individuals, but here they are measured at the level of states. Nevertheless, the only way the results might not support a relatively increased AE reporting rate among individual Republican-voting citizens is if Republican-voting citizens were less likely to report but far more likely than Democrat-voting citizens to be vaccinated in the first place or if, as the proportion of Republican-voting citizens in a state increased, the AE reporting rates among the progressively fewer Democrat-voting citizens increased at an even steeper rate. Neither possibility seems likely.

This study also has some strengths. Its results were sustained across alternative statistical modeling approaches and in multiple sensitivity analyses adjusting for population heterogeneity.

## Conclusions

The association between observation and belief runs both ways. The adage “seeing is believing” recognizes that our individual experiences inform our sense of truth, and “believing is seeing” recognizes that our preconceptions modulate what we experience in the first place. In finding that Republican-inclined states show higher COVID-19 AE reporting than Democrat-inclined states, this study suggests that Republicans are more likely to perceive or report those AEs and that Democrats are less likely to.

## Notes

Supplement 1. **eAppendix 1.** Data Sources **eAppendix 2.** Statistical Modeling **eFigure.** Association Between State-Level Influenza Vaccine AE Reporting Rates vs Political Leaning **eTable 1.** State-Level Political Inclination and Counts of Vaccine Administered and VAERS AE Reports **eTable 2.** The First Sensitivity Analysis
eReferences.

Supplement 2.
Data Sharing Statement
