# Supplementary material for: Reports of COVID-19 Vaccine Adverse Events in Predominantly Republican vs Democratic States
Source: JAMA Netw Open. 2024 Mar 29;7(3):e244177. doi: 10.1001/jamanetworkopen.2024.4177 (PMC10980960; doi:10.1001/jamanetworkopen.2024.4177)
Supplement: Supplement 1. — eAppendix 1. Data Sources eAppendix 2. Statistical Modeling eFigure. Association Between State-Level Influenza Vaccine AE Reporting Rates vs Political Leaning eTable 1. State-Level Political Inclination and Counts of Vaccine Administered and VAERS AE Reports eTable 2. The First Sensitivity Analysis eReferences. [file jamanetwopen-e244177-s001.pdf]

Supplementary Online Content

Asch DA, Luo C, Chen Y. Reports of COVID-19 vaccine adverse events in predominantly Republican vs Democratic states. *JAMA Netw Open.* 2024;7(3):e244177. doi:10.1001/jamanetworkopen.2024.4177

eAppendix 1. Data Sources
eAppendix 2. Statistical Modeling
eFigure. Association Between State-Level Influenza Vaccine AE Reporting Rates vs Political Leaning
eTable 1. State-Level Political Inclination and Counts of Vaccine Administered and VAERS AE Reports
eTable 2. The First Sensitivity Analysis
eReferences.

This supplementary material has been provided by the authors to give readers additional information about their work.

© 2024 Asch DA et al. *JAMA Network Open.*

eAppendix 1. Data Sources

Data used for this study were obtained from the following sources, accessed 07.25.2023

- The VAERS data were downloaded from https://vaers.hhs.gov/data/datasets.html.
- The US 2020 presidential election data were downloaded from the MIT Election Data and Science Lab (https://dataverse.harvard.edu/dataset.xhtml?persistentId=doi:10.7910/DVN/42MVDX).
- The influenza vaccine administration data were downloaded from the CDC Flu VaxView (https://www.cdc.gov/flu/fluvaxview/interactive-general-population.htm).
- The state male/female ratio and median age data were calculated from the American Community Survey 2020 census data from https://data.census.gov/ and the R package "tidycensus" (https://cran.r-project.org/web/packages/tidycensus/index.html).

© 2024 Asch DA et al. *JAMA Network Open.*

eAppendix 2. Statistical Modeling

The straight lines in the manuscript Figure were fitted by linear regression weighted by both sample size and residual heteroskedasticity. Specifically, a linear regression of $\frac{Y_{1i}}{n_{1i}} \sim Rep_i$ weighted by $w_i^{SS} = n_{1i}/\sum n_{1i}$ obtains residual $\hat{e}_i$. The weights by residual heteroskedasticity[1-3] are $w_i^{RH} = |\hat{e}_i|^{-1}/\sum|\hat{e}_i|^{-1}$ and the final weights are $(w_i^{SS} * w_i^{RH})/sum(w_i^{SS} * w_i^{RH})$. The calculation of R^2 only involved sample size weighting but not heteroskedasticity weighting.

The smoothing curves in the manuscript Figure were fitted by the locally estimated scatterplot smoothing (LOESS) lines. The local estimate at each point was calculated using all data and tricubic weighting of distance, and each state weighted by its total vaccine administration (R function *loess*). This smooth fitting was also applied to the FLU vaccines AE reporting, the curves are shown below, with no obvious positive nor negative association between percent of Republican in 2020 presidential election and FLU vaccine AE reporting rates.

© 2024 Asch DA et al. *JAMA Network Open.*

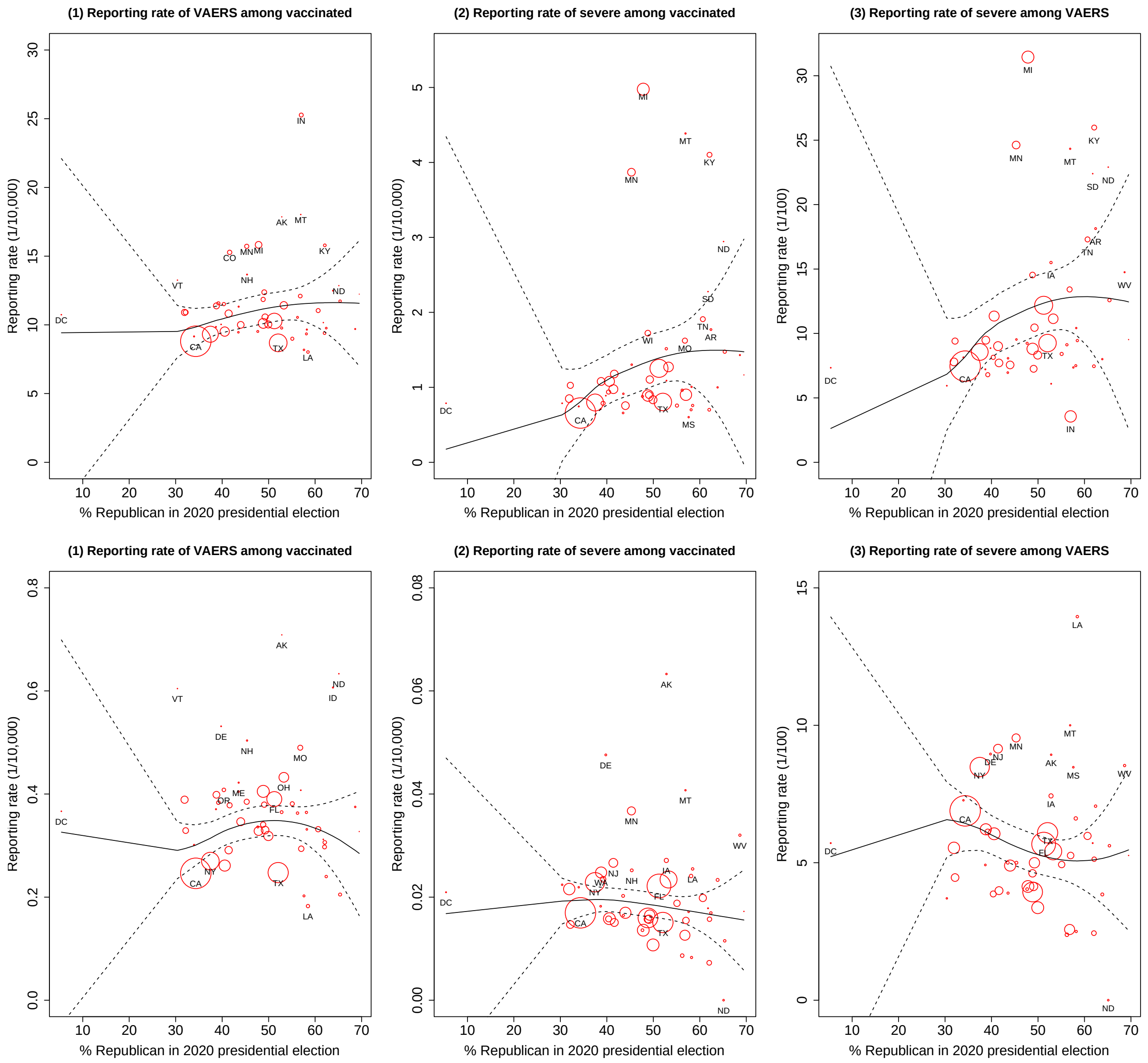

eFigure. Association Between State-Level Influenza Vaccine AE Reporting Rates vs. Political Leaning. The upper and bottom panels are for COVID and influenza vaccines respectively, and the three outcomes are: (1) any AE; (2) severe AE; (3) proportion of AE reported as severe. The smoothing curves were fitted by the locally estimated scatterplot smoothing (LOESS) method, with each state weighted by its denominators, i.e. total vaccinated in panel (1) and (2), or total VAERS reports in panel (3). Circle sizes are proportional to the number of VAERS reports of the state.

The logistic regression model in the manuscript was fit for the three outcomes separately. Here we used the first outcome, e.g. VAERS AE reports among all vaccinated, as an example to illustrate the model setting. Denote Y_{0i} and Y_{1i} as the counts of VAERS reports (numerators), and n_{0i} and n_{1i} the total number of vaccinated (denominators) for influenza and COVID19 vaccines respectively in the ith state. The logistic regression assumes $Y_{1i} \sim Binom(n_{1i}, p_{1i})$ and:

$$logit(p_{1i}) = \alpha + logit\left(\frac{Y_{0i}}{n_{0i}}\right) * \gamma + MFratio_i * \gamma_S + MedianAge_i * \gamma_A + Rep_i * \beta,$$

© 2024 Asch DA et al. *JAMA Network Open.*

where $logit(x) = \log\left(\frac{x}{1-x}\right)$, and $MFratio_i$, $MedianAge_i$, $Mandate_i$, Rep_i are state Male/Female ratio, state median age, and state 2020 Republican vote percentage, respectively. The above regression was also fit in each of the age strata 18-49, 50-64 and 65+, where the state median age variable was not included. The results are shown in Table S3. The association between political inclination and any of the three reporting rates is stronger in older populations.

As the first sensitivity analyses, we developed hierarchical logistic regression models for both COVID and influenza vaccines AE reporting. Instead of directly adjusting for the influenza vaccine reporting rate (i.e. the $logit\left(\frac{Y_{0i}}{n_{0i}}\right)$ term in the above regression), we included state-specific random effects to account for heterogeneity in baseline VAERS reporting rate. The model was
$logit(p_{ki}) = \alpha_i + MFratio_i * \gamma_S + MedianAge_i * \gamma_A + Rep_i * \beta_0 + I_{covid} * \beta_V + I_{covid} * Rep_i * \beta, k = 0,1.$
Here α_i's are state-specific random intercepts, I_{covid} is the indicator for COVID vaccines ($k = 1$) and β_V is the effect of COVID vaccines compared to influenza vaccines. The coefficient β represents the interested political effect of COVID vaccines compared to influenza vaccines. This hierarchical logistic regression was also fitted within each age strata. The results are in Table S4 and again show that the association between political inclination and any of the three reporting rates is stronger in older populations.

As another sensitivity analysis, we further performed an individual-level analysis within the VAERS reports for outcome (3), i.e. severe reports among all reported. For each of the VAERS report, while the political inclination was still state-level, the individual-level age, sex, and history of medication or allergy were now available. Vaccines other than COVID are used as reference (instead of influenza vaccines). We fit hierarchical logistic regression with state-level random intercepts. The age effect may be non-linear so we used splines (b-spline with d.f.=4, degree=3) for individual age. We also allowed the political effects to be interacted with vaccine type, sex, age and history for potential effect modifiers. The political effects are shown in Figure S1. The political effect on severe AE reports for COVID vaccines is the same significant between male and female, not significant for those with history of medication or allergy, and stronger among older individuals for COVID vaccines. The political effect is not significant for vaccines other than COVID vaccines.

© 2024 Asch DA et al. *JAMA Network Open.*

eTable 1. State-Level Political Inclination and Counts of Vaccine Administered and VAERS AE Reports
The numbers in the parentheses are number per 10,000 administered.

				COVID19 vaccines (2020-2022)			influenza vaccines (2019-2022)		
	State	Elected	% REP, 2020	# administered	# AE reports	# severe AE reports	# administered	# VAERS reports	# severe AE reports
1	DC	DEM	5.4	1,675,373	1799 (10.74)	132 (0.79)	955,039	35 (0.37)	2 (0.02)
2	VT	DEM	30.4	1,460,689	1936 (13.25)	115 (0.79)	893,138	54 (0.6)	2 (0.02)
3	MA	DEM	31.9	15,446,490	16842 (10.9)	1312 (0.85)	10,204,854	397 (0.39)	22 (0.02)
4	MD	DEM	32.2	12,330,889	13458 (10.91)	1266 (1.03)	8,175,614	269 (0.33)	12 (0.01)
5	HI	DEM	34.0	3,005,316	2752 (9.16)	224 (0.75)	1,825,541	55 (0.3)	4 (0.02)
6	CA	DEM	34.3	75,315,201	66348 (8.81)	4951 (0.66)	43,070,419	1060 (0.25)	73 (0.02)
7	NY	DEM	37.5	39,440,655	36757 (9.32)	3148 (0.8)	24,903,223	672 (0.27)	57 (0.02)
8	RI	DEM	38.7	2,475,648	2439 (9.85)	176 (0.71)	1,646,348	61 (0.37)	3 (0.02)
9	WA	DEM	38.8	14,960,189	17028 (11.38)	1614 (1.08)	9,685,925	386 (0.4)	24 (0.02)
10	CT	DEM	39.2	7,844,248	9073 (11.57)	617 (0.79)	5,112,897	196 (0.38)	12 (0.02)
11	DE	DEM	39.8	1,878,888	1885 (10.03)	167 (0.89)	1,260,775	67 (0.53)	6 (0.05)
12	OR	DEM	40.4	8,011,445	9221 (11.51)	751 (0.94)	5,073,093	207 (0.41)	8 (0.02)
13	IL	DEM	40.6	22,914,503	21799 (9.51)	2474 (1.08)	15,812,592	413 (0.26)	25 (0.02)
14	NJ	DEM	41.4	17,891,121	19361 (10.82)	1746 (0.98)	10,512,714	306 (0.29)	28 (0.03)
15	CO	DEM	41.6	10,934,708	16704 (15.28)	1288 (1.18)	7,298,841	276 (0.38)	11 (0.02)
16	NM	DEM	43.5	4,176,776	3951 (9.46)	275 (0.66)	2,471,250	100 (0.4)	5 (0.02)
17	ME	DEM	43.6	3,084,923	3494 (11.33)	282 (0.91)	1,824,402	77 (0.42)	3 (0.02)
18	VA	DEM	44.0	16,694,127	16698 (10)	1262 (0.76)	11,204,113	388 (0.35)	19 (0.02)
19	MN	DEM	45.3	10,652,270	16741 (15.72)	4122 (3.87)	7,350,429	283 (0.39)	27 (0.04)
20	NH	DEM	45.4	2,657,286	3630 (13.66)	346 (1.3)	1,985,121	100 (0.5)	5 (0.03)
21	NV	DEM	47.7	4,983,877	4744 (9.52)	438 (0.88)	2,945,311	99 (0.34)	4 (0.01)
22	MI	DEM	47.8	16,503,926	26115 (15.82)	8214 (4.98)	12,521,159	411 (0.33)	17 (0.01)
23	WI	DEM	48.8	10,548,224	12501 (11.85)	1817 (1.72)	7,634,052	260 (0.34)	12 (0.02)
24	PA	DEM	48.8	24,347,384	24620 (10.11)	2168 (0.89)	16,931,184	686 (0.41)	27 (0.02)
25	AZ	DEM	49.1	12,136,000	15015 (12.37)	1090 (0.9)	7,643,293	290 (0.38)	12 (0.02)
26	GA	DEM	49.2	15,190,054	16059 (10.57)	1678 (1.1)	10,908,409	360 (0.33)	18 (0.02)
27	NC	REP	49.9	17,465,254	17516 (10.03)	1458 (0.83)	13,056,846	416 (0.32)	14 (0.01)
28	FL	REP	51.2	38,125,650	39206 (10.28)	4778 (1.25)	21,219,768	828 (0.39)	47 (0.02)
29	TX	REP	52.1	43,771,053	38047 (8.69)	3522 (0.8)	28,512,595	706 (0.25)	43 (0.02)
30	IA	REP	52.8	5,328,027	5205 (9.77)	807 (1.51)	4,055,964	148 (0.36)	11 (0.03)
31	AK	REP	52.8	1,166,325	2083 (17.86)	127 (1.09)	790,050	56 (0.71)	5 (0.06)
32	OH	REP	53.3	18,363,803	20977 (11.42)	2337 (1.27)	13,666,128	591 (0.43)	32 (0.02)
33	SC	REP	55.1	7,839,510	7051 (8.99)	593 (0.76)	5,851,722	223 (0.38)	11 (0.02)
34	KS	REP	56.2	4,802,933	5066 (10.55)	462 (0.96)	3,471,690	126 (0.36)	3 (0.01)
35	MO	REP	56.8	9,268,645	11211 (12.1)	1504 (1.62)	7,144,006	350 (0.49)	9 (0.01)
36	MT	REP	56.9	1,661,940	2996 (18.03)	729 (4.39)	1,227,506	50 (0.41)	5 (0.04)
37	IN	REP	57.0	9,803,156	24770 (25.27)	883 (0.9)	7,759,695	228 (0.29)	12 (0.02)
38	MS	REP	57.6	3,935,818	3220 (8.18)	237 (0.6)	2,916,055	59 (0.2)	5 (0.02)
39	UT	REP	58.1	5,081,475	4747 (9.34)	356 (0.7)	3,320,935	121 (0.36)	8 (0.02)
40	NE	REP	58.2	3,259,061	3147 (9.66)	328 (1.01)	2,413,661	80 (0.33)	2 (0.01)
41	LA	REP	58.5	6,359,141	5102 (8.02)	482 (0.76)	4,711,836	86 (0.18)	12 (0.03)
42	TN	REP	60.7	9,865,390	10891 (11.04)	1884 (1.91)	7,565,949	251 (0.33)	15 (0.02)
43	SD	REP	61.8	1,515,096	1540 (10.16)	345 (2.28)	1,121,991	35 (0.31)	2 (0.02)
44	AL	REP	62.0	6,623,334	6224 (9.4)	464 (0.7)	5,516,551	164 (0.3)	4 (0.01)
45	KY	REP	62.1	6,813,118	10757 (15.79)	2795 (4.1)	5,094,430	156 (0.31)	8 (0.02)
46	AR	REP	62.4	4,388,710	4284 (9.76)	777 (1.77)	3,543,805	85 (0.24)	6 (0.02)

© 2024 Asch DA et al. *JAMA Network Open.*

47	ID	REP	63.8	2,560,677	3198 (12.49)	256 (1)	1,714,207	104 (0.61)	4 (0.02)
48	ND	REP	65.1	1,144,302	1471 (12.85)	337 (2.95)	931,496	59 (0.63)	0 (0)
49	OK	REP	65.4	6,071,981	7120 (11.73)	896 (1.48)	4,343,097	89 (0.2)	5 (0.01)
50	WV	REP	68.6	2,774,922	2691 (9.7)	397 (1.43)	2,186,789	82 (0.37)	7 (0.03)
51	WY	REP	69.5	789,353	966 (12.24)	92 (1.17)	580,635	19 (0.33)	1 (0.02)

© 2024 Asch DA et al. *JAMA Network Open.*

eTable 2. The First Sensitivity Analysis

State-level association analyses between political inclination and COVID vaccine AE reporting rate using influenza vaccines as control group. The outcomes are (1) VAERS reports among administered, (2) severe VAERS reports among administered and (3) severe reports among VAERS. The odds ratios (ORs) are estimated from the state-level hierarchical logistic regression model adjusting for vaccine type (COVID vs influenza), state male/female ratios and median age. The regression is fitted using overall and age-stratified data. State median age is not adjusted in age-stratified analyses.

Age strata	Outcome	COVID vs influenza			COVID		
		OR	95% CI	p-value	OR	95% CI	p-value
Overall (>=18 Years)	(1)	1.024	(1.004, 1.043)	0.018	0.991	(0.944, 1.04)	0.714
	(2)	1.332	(1.227, 1.447)	<0.001	1.135	(1.014, 1.271)	0.028
	(3)	1.356	(1.246, 1.477)	<0.001	1.167	(1.042, 1.308)	0.008
18-49 Years	(1)	0.989	(0.958, 1.02)	0.474	0.992	(0.941, 1.047)	0.78
	(2)	1.102	(0.956, 1.271)	0.179	1.026	(0.955, 1.103)	0.483
	(3)	1.154	(1.001, 1.332)	0.049	1.032	(0.963, 1.106)	0.37
50-64 Years	(1)	1.055	(1.015, 1.097)	0.007	1.012	(0.962, 1.064)	0.652
	(2)	1.276	(1.086, 1.499)	0.003	1.121	(1.013, 1.241)	0.028
	(3)	1.248	(1.058, 1.472)	0.009	1.122	(1.011, 1.246)	0.03
>=65 Years	(1)	1.06	(1.027, 1.094)	<0.001	0.984	(0.928, 1.043)	0.583
	(2)	1.504	(1.318, 1.717)	<0.001	1.167	(1.006, 1.352)	0.041
	(3)	1.528	(1.331, 1.754)	<0.001	1.244	(1.066, 1.452)	0.006

© 2024 Asch DA et al. *JAMA Network Open.*

eReferences.

1. White, H., 1980. A heteroskedasticity-consistent covariance matrix estimator and a direct test for heteroskedasticity. *Econometrica: journal of the Econometric Society*, pp.817-838.
2. Long, J.S. and Ervin, L.H., 2000. Using heteroscedasticity consistent standard errors in the linear regression model. *American statistician*, pp.217-224.
3. Carroll, R. J., and Ruppert, D. (1988), Transformation and Weighting in Regression, New York: Chapman and Hall.

© 2024 Asch DA et al. *JAMA Network Open.*
