# Supplementary material for: Reports of COVID-19 Vaccine Adverse Events in Predominantly Republican vs Democratic States
Source: JAMA Netw Open. 2024 Mar 29;7(3):e244177. doi: 10.1001/jamanetworkopen.2024.4177 (PMC10980960; doi:10.1001/jamanetworkopen.2024.4177)
Supplement: Supplement 2. — Data Sharing Statement [file jamanetwopen-e244177-s002.pdf]

Data Sharing Statement

Asch. Reports of COVID-19 Vaccine Adverse Events in Predominantly Republican vs Democratic States. *JAMA Netw Open*. Published March 29, 2024. doi:10.1001/jamanetworkopen.2024.4177

Data
Data available: Yes
Data types: Other (please specify)
Additional Information: All data are openly available
How to access data: All data are openly available
When available: With publication

Supporting Documents
Document types: Other (please specify)
Additional Information: Analytic approach
How to access documents: Analytic approach
When available: With publication

Additional Information
Who can access the data: All data are openly available
Types of analyses: All data are openly available
Mechanisms of data availability: All data are openly available
Any additional restrictions: all data are openly available
